# PSYCROSSWORD 1

**DOI:** 10.4103/0019-5545.33270

**Published:** 2007

**Authors:** T. V. Asokan

**Affiliations:** Asst. Professor of Psychiatry, Institute of Mental Health, Chennai - 600010, India. E-mail: tvasokan@vsnl.com

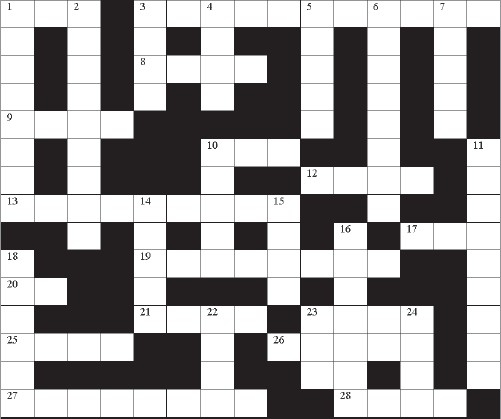



## CLUES

### Across

1. Proponent of Dhat syndrome (3)

3. He first described Anorexia Nervosa (7,4)

8. Acronym for premier organization for Substance Abuse (1,1,1,1)

9. Group of friends that will have greater influence on adolescence (4)

10. Acronym for inborn error of metabolism due to deficiency of Phenylalanine Hydroxylase (1,1,1)

12. Said to be mentally normal (4)

13. Proponent of Infantile Autism (3,6)

17. Melatonin and Light therapy are recommended for this mood disorder (1,1,1)

19. He first described Trichotillomania (8)

20. Pleasure Principle (2)

21. Proponent of Phobic Anxiety Depersonalisation Syndrome (4)

23. _ _ _ _Rage: spontaneous outbursts of motor activity and fear (4)

25. _ _ _ _ Krapelin: He highlighted Dementia Praecox (4)

26. Type of alcoholism with increased tolerance, severe withdrawal, also known as ‘malignant alcoholism’ (5)

27. ‘Birth trauma’ was his concept (4,4)

28. He advocated the ‘Guru-Chela’concept (4)

### Down

1. Proponent of Agorophobia (8)

2. He first described ‘Borderline Personality Disorder’ (9)

3. Present State Examination was compiled by him (4)

4. Marital Schism was highlighted by him (4)

5. He gave the principle of “inferiority Complex” (5)

6. Proponent of Periodic Catatonia (8)

7. Anti-Psychiatrist (5)

10. He revolutionized by unchaining the mentally ill (5)

11. Concrete and Abstract thinking were first highlighted by him (9)

14. Proponent of Myxodema Madness (5)

15. Section 375 IPC deals with _ _ _ _ (4)

16. He was the proponent of ‘Primary Obsessional Slowness’ (7)

18. Libido was first explained by him (6)

22. _ _ _ _ study: important genetic study with doubles (4)

23. General term for depressed mood (3)

24. In Greek, ’Persona” means _ _ _ _ (4)

